# Comparative outcomes of two competitive devices for retrograde closure of perimembranous ventricular septal defects

**DOI:** 10.3389/fcvm.2023.1215397

**Published:** 2023-07-05

**Authors:** Raymond N. Haddad, Zakhia S. Saliba

**Affiliations:** ^1^ Centre de Référence Malformations Cardiaques Congénitales Complexes – M3C, Hôpital Universitaire Necker-Enfants Malades, Assistance Publique—Hôpitaux de Paris, Paris, France; ^2^ Department of Pediatric Cardiology, Hotel Dieu de France University Medical Center, Saint Joseph University, Alfred Naccache Boulevard, Achrafieh, Beirut, Lebanon

**Keywords:** perimembranous, ventricular septal defect, device closure, amplatzer duct occluder, multifunctional occluder

## Abstract

**Background:**

Retrograde closure of perimembranous ventricular septal defects (pmVSDs) is a well-established procedure. However, interventionists are still looking for the best closure device.

**Methods:**

We performed a single-center retrospective review of 5-year-experience (from July 2015 to July 2020) with retrograde closure of pmVSDs using Amplatzer^TM^ Duct Occluder II (ADOII) and KONAR-MF™ VSD occluder (MFO). Deficient sub-aortic rim (SAR) (≤2.5 mm for MFO and ≤3 mm for ADOII) was an exclusion criterion in defects with a diameter ratio (right-side exit/left-side entry) > 0.5.

**Results:**

We identified 77 patients (57.1% males) with a median age of 4.3 years (IQR, 2.2–8.3) and a median weight of 16 kg (IQR, 11.2–24.5). 44 (57.1%) defects (22.7% with deficient SARs) with a median left-side defect diameter of 8.7 mm (IQR, 5.7–10) were closed with ADOIIs. 33 (42.9%) defects (51.5% with deficient SARs) with a median left-side defect diameter of 10.8 mm (IQR, 8.8–13.5) were closed with MFOs. One 7/5 MFO was removed before release and upsized to a 12/10 MFO. Implantation success rate was 100% with ADOII and 90.9% with MFO devices. Two MFOs were snare-recaptured after embolization, and one 9/7 MFO was snare-retrieved for a new onset of grade-2 aortic regurgitation that persisted afterward. Median follow-up was 3.3 years (IQR, 2.1–4.2) for ADOII and 2.3 years (IQR, 1.7–2.5) for MFO. No permanent heart block or death occurred. Freedom from left ventricular dilation was 94.62% at 36 months of follow-up. Freedom from residual shunt was 90.62% for MFO and 89.61% for ADOII at 24 months of follow-up. One 2.6-year-old patient with baseline mild aortic valve prolapse and trivial aortic regurgitation developed a grade-2 aortic regurgitation after 9/7 MFO implantation. He was treated surgically after two years without device extraction. One new grade-2 asymptomatic tricuspid regurgitation persisted at the last follow-up in the ADOII group.

**Conclusions:**

ADOII and MFO are complementary devices for effective retrograde closure of pmVSDs in children, including defects with absent or deficient SAR. ADOII is limited to smaller defects but offers a lower profile and a flexible left-side disk for better maneuverability over the aortic valve during retrograde implantation.

## 1. Introduction

**Figure 1:**
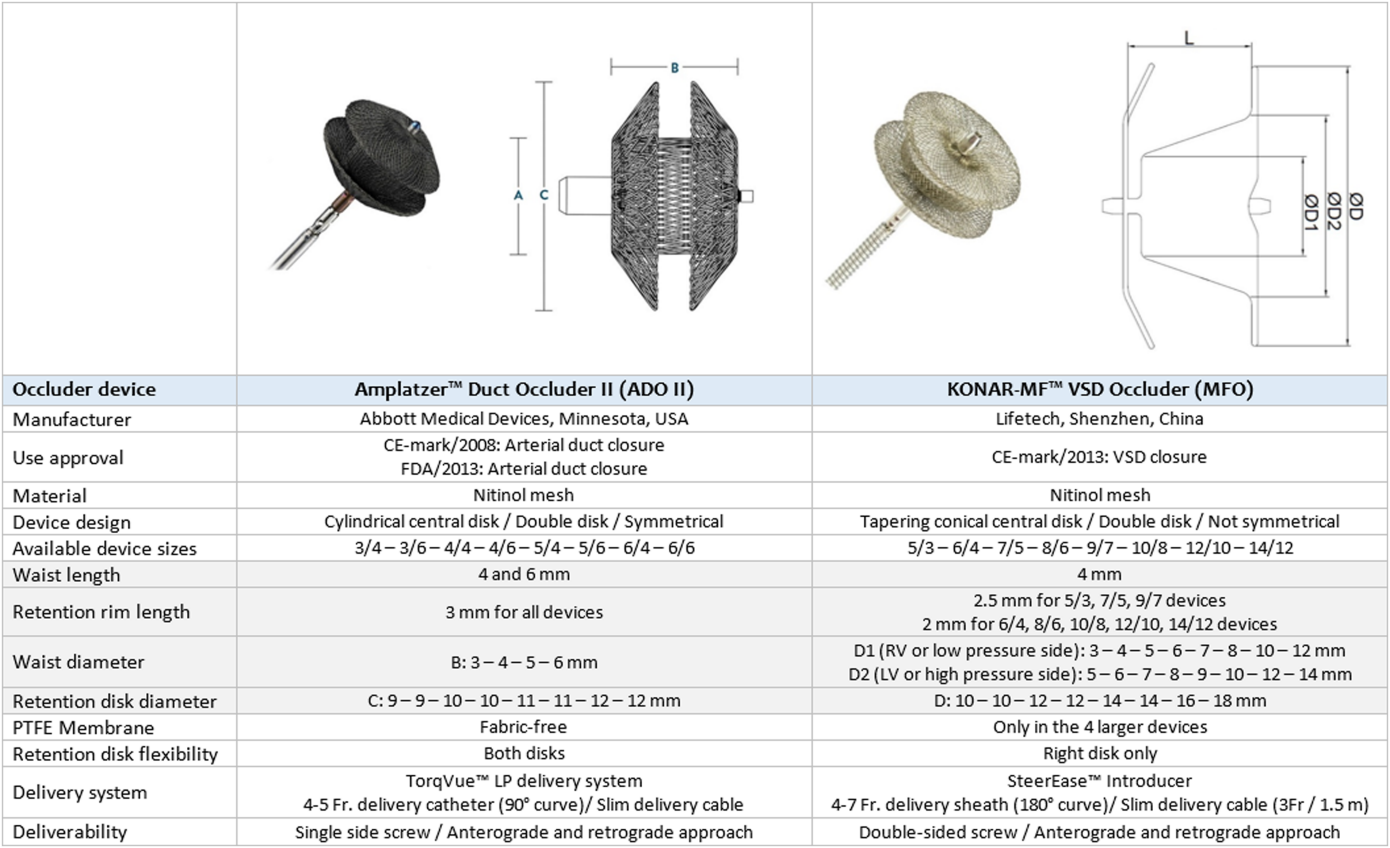
Comparative diagram between ADOII and MFO.

Device closure of perimembranous ventricular septal defects (pmVSDs) is a complex transcatheter intervention with stringent demands on device design due to challenging considerations (1–3). Interventionists have been reporting successful experiences with encouraging outcomes using various devices (1–6). As the economics of material cost, physician time, and irradiation exposure are influential, the advantages of the retrograde approach for device closure are becoming an area of interest (7–11). Amplatzer™ duct occluder II (ADOII) (Abbott Cardiovascular, USA), a device designed for arterial duct closure, and the more recent KONAR-MF™ VSD occluder (MFO) (Lifetech, China) are convenient double-disk occluders for retrograde approach with interesting characteristics (Figure 1) (9–11). The safety and short-term efficacy of these two devices for pmVSD closure have been described in separate cohorts by us and others (8–19). We expand upon these earlier findings and comprehensively evaluate the midterm outcomes of our experience with retrograde closure using these two devices. We focus on the technical considerations in device selection once the decision to intervene has been made and on the fate of encountered complications to present learning points and a comprehensive device selection protocol.

## 2. Patients and methods

### 2.1. Study design

We performed a retrospective data review of all consecutive patients with hemodynamically significant but restrictive-type pmVSDs and scheduled for an attempted retrograde transcatheter closure, using ADOII or MFO at our institution between July 2015 and July 2020. Patients were divided into two groups according to the implanted device. Standard safety and midterm outcomes were compared. All procedures contributing to this work comply with the ethical standards of the relevant national guidelines on human experimentation, and with the Helsinki Declaration of 1975, as revised in 2008. Approval from the institutional review board was obtained. Written informed consent was signed by the patients or their legal guardians to perform the procedure and to use their clinical records for eventual publication.

### 2.2. Patient selection and pre-procedure ultrasound evaluation

Patients included in this study were referred to our center for clinical evidence of hemodynamically significant left-to-right shunting but restrictive-type pmVSDs. They were scheduled for an attempted device closure after a multidisciplinary discussion. Before the procedure, patients underwent protocolized 2D transthoracic echocardiography (TTE) to evaluate pmVSD location, morphology, size, and hemodynamic relevance (20). The shunt was considered clinically significant in the case of left ventricle (LV) volume overload. We defined left heart overload as echocardiographic LV end-diastolic diameter (LVEDD) *Z*-score ≥ 2.0 (21). Left heart overload was also supported by the presence of 1) symptoms of heart failure not improving with medications, 2) failure to thrive, and or 3) recurrent respiratory infections. Few children were diagnosed with clinically relevant pmVSD after the age of 5 years and were sent directly for device closure. We assessed pulmonary arterial pressures on TTE. We also considered the history of documented infective bacterial endocarditis related to the pmVSD as an inclusion criterion.

The TTE evaluation focused on three measurements to guide the device selection that was assessed later by angiography and intra-procedural transesophageal echocardiography (TEE). We defined the defect depth as the distance between the LV entry and the RV exit point, and its measurement was more reliable on angiography than ultrasound. We measured the LV entry diameter using three views (parasternal short-axis, apical 3-chambers view, and subcostal LV-to-Aorta). We also used the parasternal short-axis view to define the number and diameters of the right ventricle (RV) exit(s) and focused on the largest color Doppler flow diameter. We measured the sub-aortic rim (SAR) (distance from the aortic valve (AoV) annulus to the upper margin of the color flow across the pmVSD) using four views (parasternal long-axis view, apical 3-chambers, apical 5-chambers, and subcostal LV-to-Aorta). We considered SAR deficiency (≤ 2.5 mm for MFO and ≤ 3 mm for ADOII) or its absence as an exclusion criterion for retrograde closure only when the diameter ratio (RV exit/LV entry) was > 0.5. The other exclusion criteria for the intervention were: (1) ≥ moderate AoV prolapse and ≥ grade 1 aortic regurgitation (AR) (22), (2) left or right ventricular outflow tract obstruction, (3) pulmonary arterial hypertension, (4) active infection, contraindication to acetylsalicylic acid, or heparin, and body weight <8 kg. No advanced imaging modalities were needed for the diagnosis or procedure planning.

### 2.3. Device selection protocol

The choice of the device was guided by four measurements: 1) the largest LV entry diameter (measured on TEE or LV angiogram at the end of diastole), 2) the shortest SAR length (measured on TEE or angiography), 3) the largest RV exit diameter (measured on TEE), and 4) defect depth (measured on angiography).

The ADOII device was inherently limited to defects with LV entry diameter <10–11 mm and RV exit diameter ≤5.5 mm. Device choice was also governed by device availability over the study period. From July 2015 to June 2018, MFO was not commercially available outside China, and small-to-medium-sized defects were closed with ADOII. In June 2018, MFO was introduced to the armamentarium and remained available in our center until July 2020. During that period, defects anatomically eligible for either device (i.e., LV entry diameter <10–11 mm and RV exit diameter ≤5.5 mm) were closed with ADOII or MFO based on the operator choice. Larger defects were exclusively closed with MFO devices.

Only 4 mm long ADOII devices were used for this procedure. In defects with sufficient SARs, the diameter of ADOII central waist was selected 1–2 mm larger than the RV exit diameter to keep the left-side retention disk (LRD) larger than the LV entry diameter. In these defects, the D2 waist diameter of the MFO was selected equal to or 1 mm larger than the LV entry diameter (Figure 1). On the other hand, in defects with absent or deficient SARs and diameter ratio (RV exit/LV entry) ≤ 0.5, the LRD diameter of both devices was chosen equal to or 1 mm larger than the LV entry diameter. In eligible cases, the device was oversized by one size in defects with deep aneurysm (≥ 7 mm) and/or diameter ratio (RV exit/LV entry) > 0.5, taking into account that the device will shrink if elongated. The institutional device selection protocol for both devices is outlined in Tables 1, 2.

**Table 1:** Institutional device selection protocol for KONAR-MF™ VSD occluder.

KONAR-MF™ VSD occluder		Deficient SAR* + RVE/LVE diameter ratio ≤ 0.5	Sufficient SAR**	
			RVE/LVE diameter ratio ≤ 0.5	RVE/LVE diameter ratio > 0.5 or deep aneurysm
Left-side D2—Right-side D1	LRD (D)	LV entry	LV entry	
5–3	10	9–10 mm	4–5 mm	3–4 mm
6–4	10	9–10 mm	5–6 mm	4–5 mm
7–5	12	11–12 mm	6–7 mm	5–6 mm
8–6	12	11–12 mm	7–8 mm	6–7 mm
9–7	14	13–14 mm	8–9 mm	7–8 mm
10–8	14	13–14 mm	9–10 mm	8–9 mm
12–10	16	15–16 mm	11–12 mm	10–11 mm
14–12	18	17–18 mm	13–14 mm	12–13 mm

LRD, left-side retention disk; LV, left ventricle; RV, right ventricle; RVE/LVE, right ventricular exist/left ventricular entry; SAR, sub-aortic rim.

* ≤2.5 mm.

** >2.5 mm.

**Table 2:** Institutional device selection protocol for Amplatzer™ duct occluder 2.

Amplatzer™ duct occluder II		Deficient SAR* + RVE/LVE diameter ratio ≤ 0.5		Sufficient SAR**			
				RVE/LVE diameter ratio ≤ 0.5		RVE/LVE diameter ratio > 0.5 or deep aneurysm	
Waist Diameter- Length	LRD	LV entry	RV exit	RV exit	LV entry	RV exit	LV entry
3–4	9	8–9 mm	<3 mm	≤2 mm	≤8 mm	<2 mm	≤7 mm
4–4	10	9–10 mm	<4 mm	≤3 mm	≤9 mm	<3 mm	≤8 mm
5–4	11	10–11 mm	<5 mm	≤4 mm	≤10 mm	<4 mm	≤9 mm
6–4	12	11–12 mm	≤5.5 mm	≤5.5 mm	≤11 mm	<5 mm	≤10 mm

LRD, left-side retention disk; LV, left ventricle; RV, right ventricle; RVE/LVE, right ventricular exist/left ventricular entry; SAR, sub-aortic rim.

* ≤3 mm.

** >3 mm.

### 2.4. Interventional procedure

We performed all interventions under general anesthesia, fluoroscopy, and TEE guidance. We combined LV angiography with intraoperative TEE to delineate the defect anatomy. We crossed the defects from the LV side and implanted the device retrogradely as previously described (10, 11). Before device release, we controlled residual shunting, valvular function, and proper device position on TEE. We also performed a hand-dye injection in the ascending aorta to assess device non-interference with AoV function. We considered the implantation successful when the device was implanted stably into position until hospital discharge (i.e., no elective or emergent device retrieval for embolization, migration, or severe complication). We prescribed all patients 6 months of daily acetylsalicylic acid and bacterial endocarditis prophylaxis.

### 2.5. Follow-Up protocol

Outpatient follow-up visits were scheduled for one week then 1, 3, 6, and 12 months after the procedure and yearly thereafter. Standard adverse events were closely monitored based on detailed physical examination, TTE, and electrocardiogram. We performed electrocardiogram-Holter monitoring (24 h) when judged clinically indicated (i.e., conduction abnormalities on electrocardiogram and/or symptoms of syncope). At 6-month follow-up, acetylsalicylic acid therapy and bacterial endocarditis prophylaxis were discontinued in patients with complete closure. We reviewed the patients' records and patients who skipped their last scheduled follow-up were called for clinical consultation.

### 2.6. Statistical analyses

Statistical analyses were performed using SPSS, Version 22.0 for Macintosh (IBM, Armonk, NY, USA). Categorical variables were reported as frequency and percentage and continuous variables were represented as median with interquartile range (IQR). The normality of measurements was assessed using Shapiro–Wilk test. Statistical analyses for continuous variables were conducted using Mann–Whitney *U* and by chi-square test and Fisher's exact test for categorical variables as appropriate. Kaplan-Meier univariate analysis was used to estimate freedom from the residual shunt and LV dilation. A *p*-value < 0.05 was considered statistically significant. All reported *P* values are two-sided.

## 3. Results

### 3.1. Patient demographics

We identified 77 patients (57.1% males) with a median age of 4.3 years (IQR, 2.2–8.3) and a median weight of 16 kg (IQR, 11.2–24.5). The demographics of the two groups of patients are detailed in Table 3. Overall, 90.9% of the patients had dilated LVs with a median LVEDD *z*-score of 3.11. Two patients treated with ADOII devices had a history of infective endocarditis. At the time of the intervention, 44.2% of patients were non-responders to heart failure medical therapy (ADOII, *n* = 20, MFO, *n* = 14), 42.9% of patients were failing to thrive (ADOII, *n* = 20, MFO, *n* = 13), and 16.9% (ADOII, *n* = 3, MFO, *n* = 10) of patients had recurrent respiratory infections.

**Table 3:** Overall demographic and procedural characteristics.

	Total, *n *= 77	ADOII, *n *= 44	MFO, *n *= 33
Male, *N* (%)	44 (57.1)	27 (61.4)	17 (51.5)
Age (years), median (IQR)	4.3 (2.2, 8.3)	3.7 (2.2, 7)	5.3 (2.3, 9.6)
Weight (kg), median (IQR)	16 (11.2, 24.5)	15 (11, 22.7)	17 (11.7, 26.7)
BSA (m^2^), median (IQR)	0.67 (0.51, 0.91)	0.64 (0.5, 0.87)	0.7 (0.53, 0.98)
Age groups, *N* (%)			
<1 year	3 (3.9)	2 (4.5)	1 (3)
1–<5 years	40 (51.9)	26 (59.1)	14 (42.4)
5–<10 years	23 (29.9)	12 (27.3)	11 (33.3)
10–<15 years	8 (10.4)	3 (6.8)	5 (15.2)
≥15 years	3 (3.9)	1 (2.3)	2 (6.1)
Weight groups, *N* (%)			
8–<15 kg	33 (42.9)	21 (47.7)	12 (36.4)
15–<30 kg	32 (41.6)	18 (40.9)	14 (42.4)
30–<50 kg	6 (7.8)	4 (9.1)	2 (6.1)
≥50 kg	6 (7.8)	1 (2.3	5 (15.2)
Down syndrome, *N* (%)	4 (5.2)	1 (2.3)	3 (9.1)
Associated congenital heart defects, *N* (%)	11 (14.3)	3 (6.8)	8 (24.2)
Indication for closure, *N* (%)			
Left chamber enlargement	70 (90.9)	39 (88.6)	31 (93.9)
LVEDD (mm), median (IQR)	41.5 (38, 45)	40 (38, 44)	43 (39, 49)
LVEDD Z-score^a^, median (IQR)	3.11 (2.25, 3.94)	3 (2.15, 3.33)	3.3 (2.4, 4.1)
History of infective endocarditis	2 (2.6)	2 (4.5)	--
Sub-aortic rim, *N* (%)			
Deficient^b^	27 (35.1)	10 (22.7)	17 (51.5)
Sufficient	50 (64.9)	34 (73.3)	16 (48.5)
LV entry (mm)^c^, median (IQR)	9.5 (7.5, 12)	8.7 (5.7, 10)	10.8 (8.8, 13.5)
RV exit (mm), median (IQR)	4 (3.4, 5)	3.5 (3, 4.2)	5 (4.5, 6)
Diameter ratio (RV exit/LV entry), median (IQR)	0.46 (0.38, 0.57)	0.45 (0.34, 0.61)	0.47 (0.38, 0.54)
Diameter ratio (RV exit/LV entry) > 0.5, *N* (%)	27 (35.1)	15 (34.1)	12 (36.4)
Number of RV exits, median (IQR)	2 (1, 2.8)	1 (1, 2.8)	2 (1, 2.8)
Pulmonary-Systemic Flow Ratio (Qp/Qs), median (IQR)	2.3 (2, 2.5)	2.3 (2, 2.4)	2.4 (2, 2.5)
Device type (without PTFE membrane), *N* (%)	57 (74)	44 (100)	13 (39.4)
Sheath in-out time (min), median (IQR)	55 (45, 68.77)	50 (40, 60)	60 (50, 70)
Fluoroscopy time (min), median (IQR)	8.7 (6.2, 14.4)	8.9 (7.3, 14.4)	7.3 (5.2, 14.8)
Total DAP (Gy.cm²), median (IQR)	11.4 (6.5, 22.8)	10.8 (6.6, 18.9)	14.1 (5.8, 34.4)
*K*_ar_ (mGy), median (IQR)	140 (82, 315.3)	133 (85.5, 284)	175 (75, 362)
Successful implantation, *N* (%)	74 (96.1)	44 (100)	30 (90.9)
Device embolization, *N* (%)	2 (2.6)	–	2 (6.1)
Elective device retrieval, *N* (%)	1 (1.3)	–	1 (3)
Follow-up duration (years), *n *= 72			
Median (IQR)	2.5 (1.8, 3.9)	3.3 (2.1, 4.2)	2.3 (1.7, 2.5)
Total range	0.8–5.9	0.8–5.9	1.2–2.9
Persistent complications at the latest follow-up, *N* (%)			
Trivial residual shunt, *n *= 74	9 (12.2)	5 (11.4)	4 (13.3)
Valvular disturbances, *n *= 75	6 (8)	4 (9.1)	2 (6.4)

ADOII, Amplatzer™ duct occluder II; MFO, KONAR-MF™ VSD occluder; BSA, body surface area; DAP, dose area product; IQR, interquartile range; *K*_ar_, cumulative air kerma at the patient entrance reference point; LV, left ventricle; LVEDD, left ventricle end-diastolic diameter; PTFE, polytetrafluoroethylene; RV, right ventricle; TEE, trans-oesophageal echocardiography.

^a^ *Z*-score are calculated based on data from Kampmann C, et al. Heart. 2000.

^b^ ≤2.5 mm for MFO and ≤3 mm for ADOII.

^c^ Average of angiographic and TEE measurements.

### 3.2. Cardiac catheterization

The median pulmonary-systemic flow ratio (Qp/Qs) was 2.3 (IQR, 2–2.5). No patient had pulmonary arterial hypertension or required pulmonary vasoactive evaluation before device closure. For attempted closure, 44 (57.1%) ADOII and 33 (42.9%) MFO devices were used. Implanted ADOII devices were 6/4 (*n* = 22, 50%), 5/4 (*n* = 11, 25%), 4/4 (*n* = 10, 22.7%), and 3/4 (*n* = 1, 2.3%). Implanted MFO devices were 14/12 (*n* = 3, 9.1%), 12/10 (*n* = 5, 15.1%), 10/8 (*n* = 4, 12.1%), 9/7 (*n* = 6, 18.2%), 8/6 (*n* = 7, 21.2%), 7/5 (*n* = 3, 9.1%), and 6/4 (*n* = 5, 15.2%). One 7/5 MFO was removed before release and upsized to a 12/10 MFO. There was no switch from one device to another or other cases of device change for a larger or smaller one. The overall anatomical characteristics of the defects are detailed in Table 3.

From July 2015 to June 2018, 25/77 (32.5%) defects (16% with deficient SARs) with a median LV diameter of 8.5 mm (IQR, 4.2–10) were closed with ADOII. From June 2018 to July 2020, 34/77 (44.1%) defects (38.2% with deficient SARs) with a median LV diameter of 9 mm (IQR, 6.5–10.4) were closed with 15 MFO and 19 ADOII while 18/77 (23.4%) larger defects (55.5% with deficient SARs) with a median LV diameter of 13.3 mm (IQR, 11.8–15.3) were closed with MFO devices.

#### 3.2.1. Patients with defects anatomically eligible for both devices (i.e. LV entry diameter <10–11 mm and RV exit ≤5.5 mm)

The demographic and procedural characteristics of the subgroup of 59 patients with defects anatomically eligible for both devices are detailed in Table 4. Patients who received MFO devices (median age of 2.2 years) were younger than those who received ADOII devices (median age of 3.7 years) (*p* = 0.022). The median RV exit diameter was 3.5 mm (IQR, 3–4.2 mm) for defects closed with ADOII and 4 mm (IQR, 4–5 mm) for those closed with MFO (*p* = 0.015). The distribution of the diameter ratio (RV exit/LV entry) was almost identical in both groups with no statistical difference. 7/15 (46.7%) defects closed with MFO had deficient SARs compared to 10/44 (24.4%) defects closed with ADOII (*p* = 0.188). Procedure and fluoroscopy time as well as irradiation exposure did not vary significantly between the two groups.

**Table 4:** Demographic and procedural characteristics of 59 patients with defects anatomically eligible for both devices *(i.e. LV entry diameter <10–11 mm and RV exit* ≤*5.5 mm*).

	Total, *n *= 59	ADOII, *n *= 44	MFO, *n *= 15	*p*-Value
Male, *N* (%)	33 (55.9)	27 (61.4)	6 (40)	0.229^a^
Age (years), median (IQR)	3.3 (2, 6.5)	3.7 (2.2, 6.9)	2.2 (1.2, 3.3)	**0.022** ^b^
Weight (kg), median (IQR)	13 (10.8, 21)	15 (11, 22.7)	12 (10, 15)	0.073^b^
Deficient sub-aortic rim^d^, *N* (%)	17 (30.4)	10 (24.4)	7 (46.7)	0.188^a^
LV entry (mm)^e^, median (IQR)	9 (6.5, 10.4)	8.7 (5.7, 10)	9 (7.5, 10.5)	0.335^b^
RV exit (mm), median (IQR)	4 (3, 4.9)	3.5 (3, 4.2)	4 (4, 5)	**0.015** ^b^
Diameter ratio (RV exit/LV entry), median (IQR)	0.47 (0.38, 0.58)	0.45 (0.34, 0.61)	0.48 (0.38, 0.54)	0.578^b^
Diameter ratio (RV exit/LV entry) > 0.5, *N* (%)	22 (37.3)	15 (34.1)	7 (46.7)	0.537^b^
Implantation time, *N* (%)				
July 2015–June 2018	25 (42.4)	25 (56.8)	--	--
June 2018–July 2020	34 (57.6)	19 (43.2)	15 (100)	
Sheath in-out time (min), median (IQR)	52.5 (45, 60)	50 (40, 60)	55 (50, 70)	0.075^b^
Fluoroscopy time (min), median (IQR)	8.7 (6.8, 13.5)	8.9 (7.3, 14.4)	7.3 (5.9, 11.5)	0.424^b^
Total DAP (Gy.cm²), median (IQR)	10 (6.1, 17.8)	10.8 (6.6,18.9)	7.9 (4, 14.5)	0.173^b^
*K*_ar_ (mGy), median (IQR)	126 (80, 257)	133 (85, 284)	106 (54, 193)	0.306^b^
Successful implantation, *N* (%)	57 (96.6)	44 (100)	13 (86.7)	0.061^c^
Device embolization, *N* (%)	1 (1.7)	--	1 (6.7)	--
Elective device retrieval, *N* (%)	1 (1.7)	--	1 (6.7)	--
Persistent complications at the latest follow-up, *N* (%)				
Trivial residual shunt, *n *= 57	6 (10.5)	5 (11.4)	1 (7.7)	1^c^
Valvular disturbances, *n *= 58	6 (10.3)	4 (9.1)	2 (14.3)	0.624^c^
Grade II aortic valve regurgitation	1 (1.7)	--	1 (7.1)	--
Tricuspid valve regurgitation	5 (8.8)	4 (9.1)	1 (7.7)	1^c^
Grade I	4	3	1	
Grade II	1	1	--	

ADOII, Amplatzer™ duct occluder II; MFO, KONAR-MF™ VSD occluder; DAP, dose area product; IQR, interquartile range; *K*_ar_, cumulative air kerma at the patient entrance reference point; LV, left ventricle; RV, right ventricle. Bold values are significant *p*-values.

^a^ Chi-square test.

^b^ Mann–Whitney *U*-test.

^c^ Fisher’s exact test.

^d^ ≤2.5 mm for MFO and ≤3 mm for ADOII.

^e^ Average of angiographic and TEE measurements.

### 3.3. Post-operative evaluation and follow-up

The implantation success rate was 100% with ADOII and 90.9% with MFO devices. In two patients, the MFO device embolized to the left pulmonary artery before discharge and was snare-recaptured with no sequelae. The two patients were rescheduled for surgical VSD closure. One 9/7 MFO was snare-retrieved in a 3.4-year-old patient for a new onset of grade-2 AR that persisted afterward. Surgical AoV repair and VSD closure were proposed to the family who does not yet agree. The AR and the LV volume are stable, and the patient is still followed.

**Figure 2:**
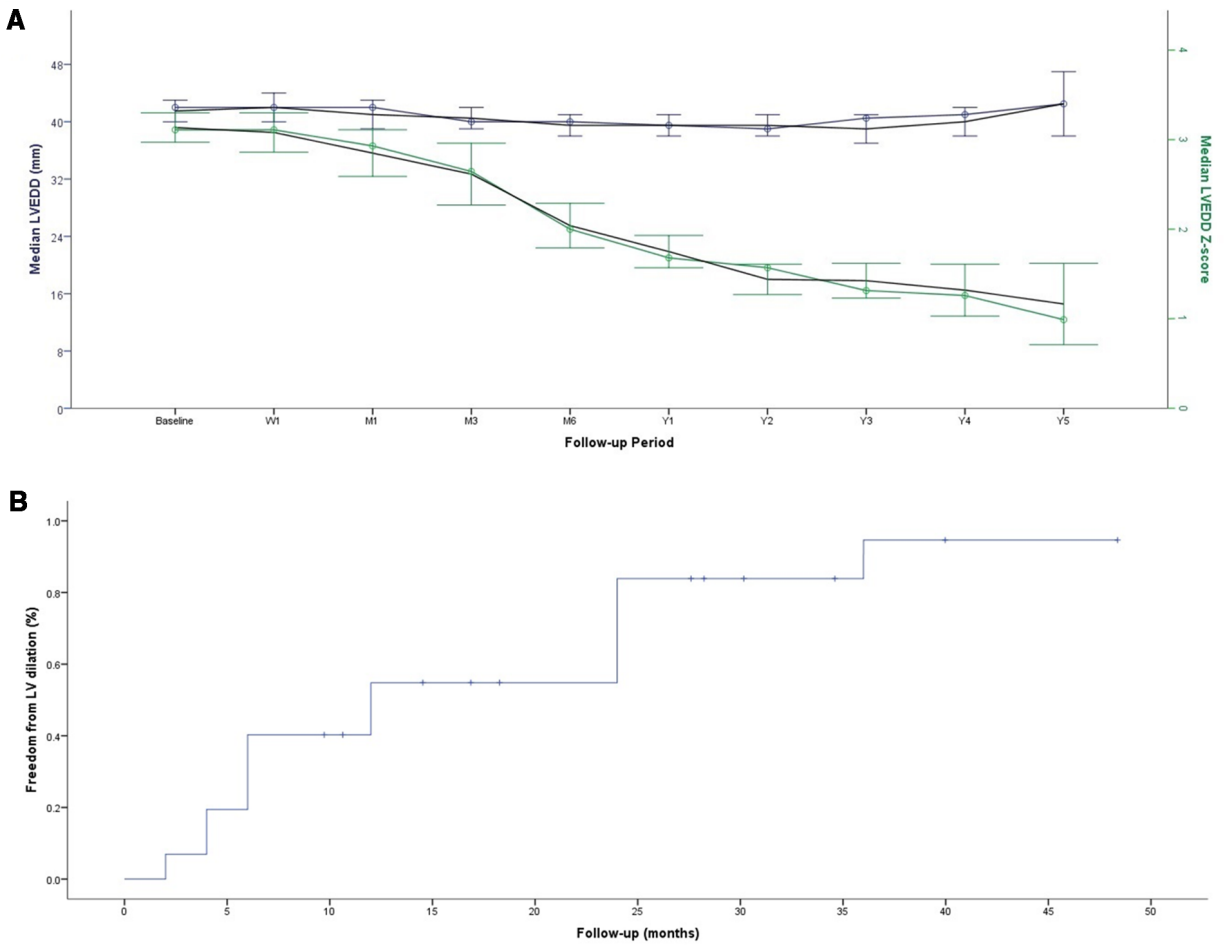
Progression of LVEDD and LVEDD *z*-score across follow-up (**A**) Kaplan-Meier curve for freedom from the persistence of LV dilation (**B**)

The median follow-up was 3.3 years (IQR, 2.1–4.2) for ADOII and 2.3 years (IQR, 1.7–2.5) for MFO devices. No permanent heart block or death occurred. One 13-year-old patient with 14/12 MFO experienced transient heart block and did not require any treatment over two years of follow-up. One 3.4-year-old patient with 6/4 ADOII had electrocardiogram-Holter monitoring for isolated premature ventricular contractions. Anticongestive drugs were stopped within one month postoperatively in all patients initially requiring this therapy. All children with failure to thrive caught up to normal growth curves. The progression of LVEDD and LVEDD z-scores across follow-up is shown in Figure 2A. Freedom from LV dilation progressively increased across follow-up, from an estimate of 83.78% at 24 months of follow-up to an estimate of 94.62% at 36 months of follow-up (Figure 2B).

**Figure 3:**
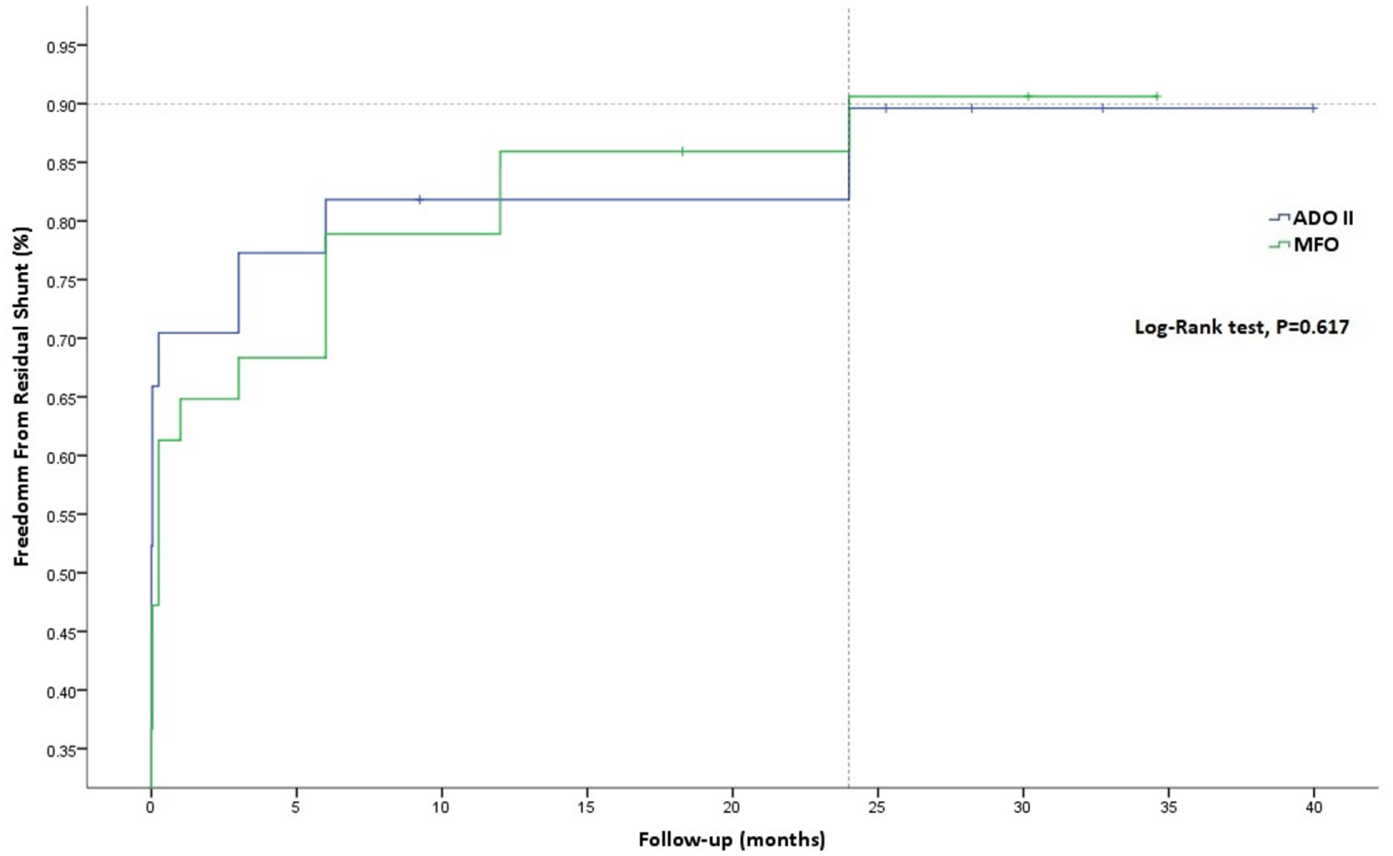
Kaplan-Meier curves for freedom from residual shunt.

**Figure 4:**
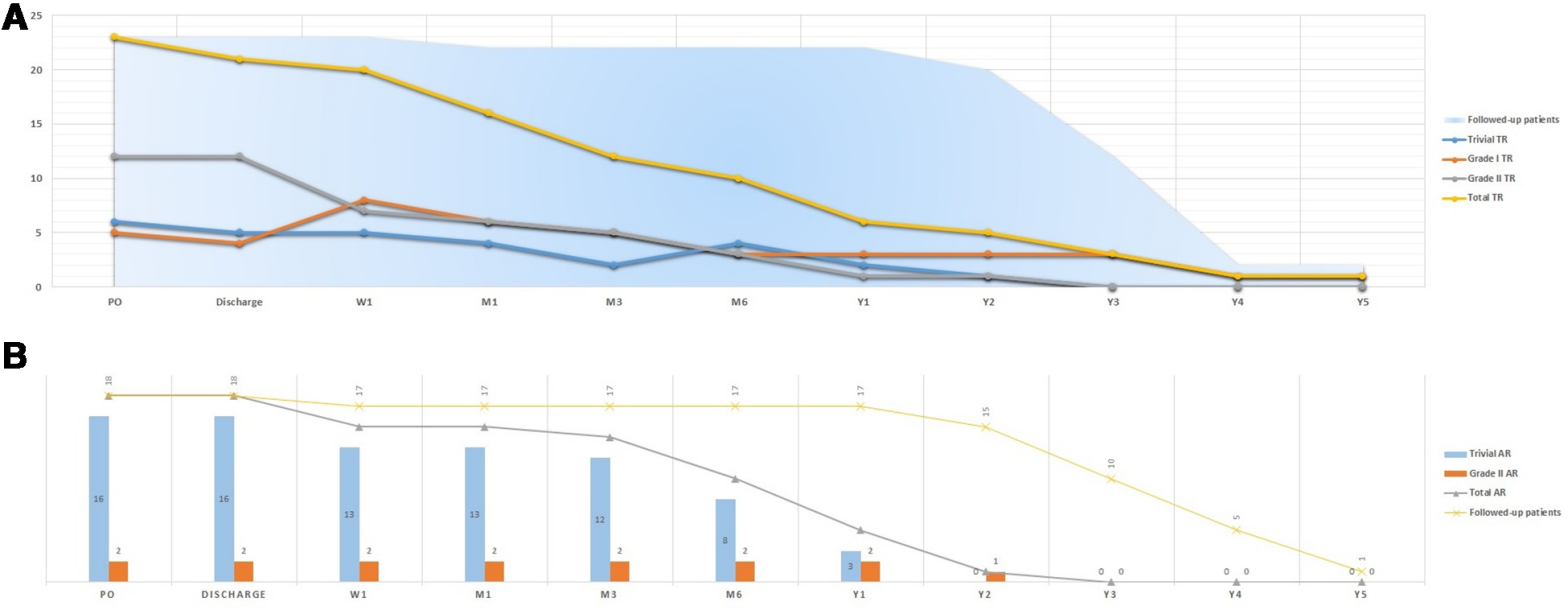
Progression of different grades of new-onset postoperative tricuspid (**A**) and aortic (**B**) valve regurgitations across follow-up.

Similarly, freedom from residual shunt progressively increased across follow-up, stabilizing at an estimate of 90.62% for MFO and 89.61% for ADOII devices (*p* = 0.617) at 24 months of follow-up (Figure 3). Overall, nine (12.2%) residual shunts (ADOII, *n* = 5, and MFO, *n* = 4) were persistent at last follow-up and were classified trivial with no hemodynamical significance. Progression of different grades of new-onset postoperative tricuspid and AoV regurgitations across follow-up is summarized in Figure 4A,B, respectively. Overall, four grade I (i.e., physiological) and one grade 2 tricuspid regurgitations (TR) were persistent at last follow-up. The five TR were clinically well tolerated and did not require intervention. One 2.6-year-old patient with baseline mild AoV prolapse and trivial AR developed a grade 2 AR after implantation of a 9/7 MFO device. He required surgical valvuloplasty (without device extraction) after 2 years with excellent results.

## 4. Discussion

The management of children with hemodynamically significant but restrictive-type pmVSD remains controversial with no clear guidelines (23). These defects are managed either by watchful waiting or by closure, depending on the clinical symptoms and practice patterns at each center. On the one hand, “prophylactic” closure is often advocated to avoid late LV dysfunction related to cardiac volume overload. However, this complication has not been well-described and some centers consider that the benefits may not outweigh the risks of surgical or percutaneous closure (23–25). On the other hand, watchful waiting is supported by the good tolerance of the shunt after one year of age (26–28). Another argument in favor of watchful waiting is that device closure has been exposed to major challenges since the failure of the asymmetrical Amplatzer Membranous VSD Occluder device (29, 30). Despite the absence of a device dedicated to this procedure, interventionists kept on testing existing devices in an off-label fashion. Published data confirmed the feasibility, safety, and efficacy of this technique (1–6). However, the specific challenges related to the perimembranous location of the defect made the intervention complex, and device-related complications were not infrequent pushing interventionists to abandon some of the devices (20). Cumulative experience showed that ADOII and the more recently introduced MFO both appear to be adequate double-disk devices for this intervention, particularly for retrograde implantation (8–19). Herein, we present a comprehensive comparison of the outcomes of these two devices in retrograde pmVSD closure and detail the fate of encountered complications.

### 4.1. Residual shunt

The goal of this procedure is to eliminate the intracardiac shunt and the LV dilation and not to transform a restrictive pmVSD into a more restrictive one and thereby increasing the risk of device-related endocarditis. From this perspective, any residual shunt even when classified as trivial should be considered a “partial failure” of the procedure even if LV dilation disappears with time. Device fabric and the presence of a polytetrafluoroethylene membrane have no direct effect on shunt closure but rather on fastening closure when the properly selected device self-center to an optimal position (31). Earlier in our experience and before the introduction of MFO to the armamentarium, we were aiming to close the largest RV exit rather than the LV entry by oversizing the ADOII device according to the RV exit (12, 19). This protocol was favored by our decision to avoid implanting larger Amplatzer devices (i.e., Amplatzer duct occluder I and Amplatzer Muscular VSD Occluder) according to the LV entry, particularly after encountering serious complications with both of these large devices (11). This approach was suitable in defects with sufficient SAR and a diameter ratio (RV exit/LV entry) close to or above 0.5 in which ADOII will be anchored without necessarily having a cone-shaped defect. The difficulty in choosing the proper ADOII device was seen in defects with deficient or absent SAR and a diameter ratio (RV exit/LV entry) close to 0.5 where properly closing the RV exist will lead to a large LRD that will be bigger than the LV entry and might interact with the AoV. When we started implanting MFO devices, we thought that the cone-shaped device might be better applied to the RV exit and that the additional high-pressure disk will ensure complete closure. However, we rapidly found out that the odds of facing a residual shunt are higher if the LV entry is not properly closed. We encountered four cases of persistent residual shunts in the MFO group. The RV side diameter (D1) of the central cone was 1–2 mm larger than the RV exit in all cases. However, the left side diameter (D2) of the cone was 1 mm smaller than the LV entry diameter in the two cases with sufficient SAR, and the LRD diameter was just equal to the LV entry in the two cases with absent SAR. Likewise, after revising the five cases of persistent residual shunts in the ADOII group, we noticed that the central disk diameter was at least 1 mm larger than the RV exit. However, the diameter of the LRD was just equal to the LV entry in the three cases with sufficient SAR, and the diameter ratio (RV exit/LV entry) was equal to 0.5 in the two cases with absent SAR, leading to an oversized LRD that had to be manipulated away from the AoV, thereby destabilizing the device position within the defect. Our results showed that ADOII has only 1% more residual shunts at 24 months of follow-up when compared to MFO (Figure 3A). Having almost the same rate of residual shunts would favor the MFO device that was used to close larger defects, not accessible to ADOII, in 18/33 (54.5%) of patients.

### 4.2. Tricuspid regurgitation

Both ADOII and MFO devices have a double disk design and can be implanted retrogradely. Compared to anterograde implantation, the retrograde approach improves the control in positioning the right-side disk away from the tricuspid valve (10, 11). In addition, both devices are designed with a freely articulating right retention disk that can be well-positioned under TEE monitoring (9–12). One might expect no new-onset of TR and might question why devices were released into position if a TR has been noticed on TEE. In fact, color Doppler examination on TEE can underestimate the degree of regurgitation (32). In some cases, multiple right disk repositioning to eliminate the regurgitation was suboptimal. These attempts included: 1) partial deployment of the right retention disk in a “mushroom” shape (i.e., before taking its final shape with flat edges) within the RV as close as possible to the interventricular septum to be then completely deployed away for the tricuspid valve or 2) re-crossing the defect and trying to come out from another “better oriented” RV exit and facilitate the right disk implantation (20). In failed attempts, we ended up implanting the device while accepting a certain degree of TR hoping that the device will adapt to its position after release and the regurgitation will progressively disappear. This phenomenon was observed immediately after device release or during follow-up (Figure 4A). All persistent TR are considered iatrogenic since they are secondary to a device not properly implanted. However, surgical closure of pmVSDs frequently creates TR (33). So, accepting mild degrees of TR after device closure is reasonable, as long as it is asymptomatic and does not progress over time, which is the general experience.

### 4.3. Aortic regurgitation

Avoiding AoV injury is fundamental in retrograde closure and hand injection in the ascending aorta is essential before device release to confirm the non-interference of the LRD with the AoV (1, 7, 10, 20). The two cases of persistent grade 2 AR were seen exclusively with MFO devices. Indeed, AoV injury was facilitated by the MFO's non-articulating LRD that didn't allow us to push on the delivery cable to re-orientate the LRD in the LV away from the AoV. This maneuver, successfully used in retrogradely delivered ADOII devices, displaced the MFO's central waist solidly connected to the LRD (11, 20). In our opinion, aneurysmal pmVSDs with a deficient or absent SAR are accessible to retrograde device closure when the diameter ratio (RV exit/LV entry) is ≤ 0.5. As outlined in our protocol, in defects with absent or deficient SAR, we focused on the diameter of the LRD of both devices and chose it equal to or 1 mm larger than the LV entry diameter. This implies that the selected devices were pushed inside the aneurysm, to keep the device away from the AoV and stabilize the device at a smaller entry diameter (inside the aneurysm). One should keep in mind that the aneurysms are not always too deep and thereby the devices cannot be pushed entirely inside as foreseen.

The absence of an aneurysmal transformation of the lesion leads generally to a diameter ratio (RV exit/LV entry) > 0.5, which we considered unfavorable for retrograde closure, in particular when the SAR is deficient or absent. In these defects, the device has to be oversized by one size so that it will stably hold at the right side. This oversizing will lead to a larger LRD that is harder to cope with and avoid the AoV during the retrograde approach. In those challenging cases, the transvenous approach is more reasonable because it allows to deploy the LRD inside the defect and away from the AoV (10, 20). Moreover, 16/77 (20.8%) patients experienced transient postoperative trivial AR (Figure 4B). Excessive wire and sheath manipulations might damage the AoV or temporarily affect its function (34). This phenomenon was also evoked in the first case of AR that persisted after device retrieval and was confirmed by the surgeon in the second case of AR.

### 4.4. Complete heart block

ADOII and MFO are considered suitable devices for this procedure because they provide less radial force compared to other available occluders (8–19, 35). We believe that unwanted conduction tissue damages were controlled with the soft and flexible design of both devices along with the avoidance of unreasonable oversizing. Of all implanted devices, ADOII and MFO have so far no history of late occurrence of heart block after pmVSD closure. However, the incidence of heart block is not eliminated and rare reported cases have occurred very early after the procedure (36–38). This serious complication can occur acutely but quite unpredictably months to several years after the procedure (30, 36). Therefore, caution is always the best statement and only a longer follow-up will reveal the real risk.

### 4.5. Learning points

Together, ADOII and MFO devices cover a large variety of selected pmVSD anatomies including those with absent or deficient SAR. ADOII and small-sized MFO devices can be both effectively implanted in defects with LV entry diameter < 10–11 mm and RV exit ≤ 5.5 mm despite their differently designed central disk. Positioning the more flexible ADOII device is less difficult than the more rigid MFO device. ADOII device offers a smaller delivery system with a freely articulating LRD for better maneuverability over the AoV during retrograde delivery. On the other side, MFO offers a larger portfolio to occlude larger defects with an LV entry diameter up to 17–18 mm, a smaller 2–2.5 mm retention rim to target smaller SARs, and finally, a tapered coned-shape central disk for aneurysmal anatomies with a diameter ratio (RV exit/LV entry) ≤ 0.5. Our future implantation protocol will include ADOII as a first choice of closure device except for defects with LV entry ≥ 10–11 mm and RV exit > 5.5 mm, where the MFO is a better choice.

### 4.6. Limitations

Our device selection protocol is an *ad hoc* gathered experience. The MFO device was introduced in June 2018, therefore earlier experience with retrograde implantation of ADOII devices might have affected the encountered results with the MFO device. Our protocol for retrograde implantation excludes defects with absent or deficient SAR and diameter ratio (RV exit/LV entry) > 0.5 in which we favor the anterograde approach. Electrocardiogram-Holter monitoring was performed only when judged clinically indicated. This might be considered a limitation to the interpretation of the results concerning rhythm disturbances.

## 5. Conclusion

Retrograde approach for transcatheter closure of pmVSD in children using ADOII or MFO devices is safe and effective with encouraging midterm outcomes. ADOII device is inherently limited to smaller defects but offers a lower profile and a flexible left-side disk to avoid AoV injury or interference during retrograde implantation. Both MFO and ADOII should be considered complementary devices in the armamentarium to tackle a large variety of selected pmVSD anatomies.

## Data Availability

The raw data supporting the conclusions of this article will be made available by the authors, upon request, to any qualified researcher.
